# Short‐term direct contact with soil and plant materials leads to an immediate increase in diversity of skin microbiota

**DOI:** 10.1002/mbo3.645

**Published:** 2018-05-29

**Authors:** Mira Grönroos, Anirudra Parajuli, Olli H. Laitinen, Marja I. Roslund, Heli K. Vari, Heikki Hyöty, Riikka Puhakka, Aki Sinkkonen

**Affiliations:** ^1^ Ecosystems and Environment Research Programme Faculty of Biological and Environmental Sciences University of Helsinki Lahti Finland; ^2^ Department of Virology School of Medicine University of Tampere Tampere Finland; ^3^ Fimlab Laboratories Pirkanmaa Hospital District Tampere Finland

**Keywords:** biodiversity hypothesis, human health, hygiene hypothesis, nature‐based materials

## Abstract

Immune‐mediated diseases have increased during the last decades in urban environments. The hygiene hypothesis suggests that increased hygiene level and reduced contacts with natural biodiversity are related to the increase in immune‐mediated diseases. We tested whether short‐time contact with microbiologically diverse nature‐based materials immediately change bacterial diversity on human skin. We tested direct skin contact, as two volunteers rubbed their hands with sixteen soil and plant based materials, and an exposure via fabric packets filled with moss material. Skin swabs were taken before and after both exposures. Next‐generation sequencing showed that exposures increased, at least temporarily, the total diversity of skin microbiota and the diversity of Acidobacteria, Actinobacteria, Bacteroidetes, Proteobacteria and Alpha‐, Beta‐ and Gammaproteobacteria suggesting that contact with nature‐based materials modify skin microbiome and increase skin microbial diversity. Until now, approaches to cure or prevent immune system disorders using microbe‐based treatments have been limited to use of a few microbial species. We propose that nature‐based materials with high natural diversity, such as the materials tested here, might be more effective in modifying human skin microbiome, and eventually, in reducing immune system disorders. Future studies should investigate how long‐term changes in skin microbiota are achieved and if the exposure induces beneficial changes in the immune system markers.

## 1 INTRODUCTION

Immune‐mediated diseases, such as asthma, allergy, type I diabetes, and inflammatory bowel diseases (IBD), have increased during the last decades in urban environments (Lerner, Jeremias, & Matthias, 2015; Okada, Kuhn, Feillet, & Bach, 2010). In developed countries, as high as 21% prevalence for asthma (To et al., 2012), 8%–35% for food allergies (Osborne et al., 2012) and 5% for other autoimmune diseases (Hayter & Cook, 2012) have been reported. In United States alone, it has been estimated that the annual costs for one individual immune‐mediated disease may range from 1 to 20 billion US dollars (e.g., AARDA, 2011). The hygiene hypothesis and its extensions (Noverr & Huffnagle, 2005; Rook et al., 2004; Strachan, 1989) postulate that the reason for the increased prevalence of these diseases is the increased hygiene level in our everyday life. High hygiene level and modern urban life‐style have led, for example, to decreased exposure to microorganisms and parasites and increased perturbations of microbiota, for example, due to extensive use of antibiotics. These changes may affect the normal development of immune system in early childhood (Stiemsma, Reynolds, Turvey, & Finlay, 2015). Most recently, the hygiene hypothesis has been extended to a biodiversity hypothesis (von Hertzen, Hanski, & Haahtela, 2011), which suggests that the rapid global biodiversity decline is related to the increase in immune‐mediated diseases: the decreased microbial diversity in the urban living environment contributes to the reduction in natural exposure to microorganisms (Parajuli et al., 2018) and prevent the natural development of immune system.

Hygiene and biodiversity hypotheses have gained support in numerous studies. These studies provide at least three types of evidence: (1) reduced diversity of personal microbiota (e.g., stool or skin microbiota) is associated with many immune‐mediated diseases (Hanski et al., 2012; Manichanh, 2006; Scher et al., 2015), (2) characteristics of the living environment, such as the level of urbanization, are associated with the prevalence of these diseases (Kondrashova, Seiskari, Ilonen, Knip, & Hyöty, 2012; Kondrashova et al., 2005; Ruokolainen et al., 2015) and (3) microbial diversity in the living environment is associated with these diseases (Ege et al., 2012; Valkonen, Wouters, Täubel, Rintala, & Lenters, 2015). An example of the first type is provided by Scher et al. (2015), who found that gut microbiota in patients with psoriatic arthritis and skin psoriasis was less diverse compared to that in healthy individuals. Kondrashova et al.'s (2005) study represents the second type of evidence as they found that the incidence of Type 1 diabetes was sixfold higher in the more urbanized Eastern Finland with a high hygiene level compared to the adjacent Russian Karelia although the frequency of the predisposing genotypes did not differ between the two populations. Ege et al.'s (2012) study is an example of the third type: they found that, compared to a reference group, children living on farms had a lower prevalence of asthma and atopy coinciding with a higher microbial diversity in the dust collected from their homes.

Even though many studies have found that the total microbial diversity matters (Ott et al., 2004; de Paiva et al., 2016; Scher et al., 2015), others have identified certain microbial groups to be more important. For example, patients with Crohn's disease had lower diversity of phylum Firmicutes in their stool (Manichanh, 2006), infants with eczema had lower diversities of phyla Bacteroidetes and Proteobacteria in stool (Abrahamsson et al., 2012) and adolescents with atopy had lower diversity of Gammaproteobacteria on skin (Hanski et al., 2012) compared to those of healthy individuals.

Based on the hygiene and biodiversity hypotheses it can be expected that increasing the exposure to diverse and natural microbial communities would diversify and change the composition of human microbiota and that this change would reduce the risk of immune‐mediated diseases. Surprisingly, although several exposure studies using separate bacterial strains have been conducted (Stiemsma et al., 2015), diverse natural material has hardly been tested experimentally and, to our knowledge, never for humans. Soils including composted gardening materials host especially diverse microbial communities (Yu et al., 2015) and thus increasing contact with such soils for urban citizens could provide a means for increasing the diversity of their microbiota and further decrease the prevalence of asthma and atopies (von Hertzen & Haahtela, 2006). This is especially significant in the light of our earlier findings, which suggest that urbanization and pollution could lead to changes in soil microbiota and urbanization reduces microbial transfer indoors (Parajuli et al., 2017, 2018).

Here, we used two simple experimental setups to study whether a short‐time contact with natural materials can change bacterial diversity on human skin. We concentrated on skin microbiota, as it has complex interactions with immune system; for example, commensal microbes can promote immune homeostasis and pathogen resistance (Chen, Fischbach, & Belkaid, 2018). Indeed, several skin disorders have been linked with imbalance of skin microbiota (e.g., Rodriques Hoffman, 2017). We collected several composted gardening materials, two moss materials and one peat material from commercial soil producers and took bacterial skin swab samples from two volunteers before and after short‐time skin exposure to these materials. Hands were first exposed directly to all the raw materials to study if bacterial diversity on skin increased after exposure. Then, we selected one material, moss, modified it and put it in fabric packets to test if similar effect can still be seen with the material filled in fabricates, which can be considered more convenient to use in everyday life. In addition to the total bacterial diversity, we inspected the diversity within bacterial phyla dominating either in soils (i.e., Proteobacteria, Acidobacteria, and Actinobacteria) (Janssen, 2006) or on human skin (i.e., Actinobacteria, Firmicutes, Bacteroidetes, and Proteobacteria) (Rodrigues Hoffmann, 2017). As Proteobacteria is often the most abundant phylum in soils and the diversity of some Proteobacterial classes has been associated with immune defense (Hanski et al., 2012), we also inspected the diversity within major Proteobacterial classes (i.e., Alpha‐, Beta‐, Gamma‐ and Deltaproteobacteria). We hypothesized that direct soil contact with gardening materials, moss and peat increase skin microbial diversity immediately after the exposure. In accordance with this hypothesis, we detected an increase in total diversity as well as in the diversity of all tested taxonomic groups excluding the phylum Firmicutes. The results support the core assumptions of hygiene and biodiversity hypotheses that contact with dirt and microbially rich nature affects the human microbiota. Here, we also propose that daily microbial exposure could be tailored by designing plant and soil based materials that comprise rich microbial flora.

## 2 MATERIAL AND METHODS

### 2.1 Experimental design: Direct hand‐exposure (experiment 1)

In the first experiment, two urban volunteers tested eight composted, soil and plant based materials. Altogether 16 materials were tested. Materials tested were collected from five commercial enterprises producing gardening materials: Biolan Oy/Novarbro Oy, Humuspehtoori Oy, Kekkilä Oy, Suomen Kunttapiha/SnowWay Oy and Vapo Oy. Materials included six commercial soil products (trade names: Musta Multa, Niittymulta, Nurmikkomulta, Perennamulta, Puistomulta, Viljelymulta), five composted soil enrichment products which are either sold directly for end users or used as raw material in other products, two different kind of forest turfs (i.e., transferrable forest floor) and two moss materials and one peat material gathered from pristine Finnish peat bogs. The composted soil enrichment products were produced from animal dung (e.g., horse and chicken dung), deciduous leaf litter, plant debris and sludge from wastewater treatment plant. The ingredients of the commercial soil products include composted dung, sludge and plant materials, peat, wood mulch and mineral soils. Products were composted and controlled according to E.U. regulations. We used non‐identifiable codes for the materials to follow the guidelines of agreements with collaborators. Volunteers rubbed their hands in a test material for 20 s, washed their hands without soap in tap water for 5 s, and dried the hands with paper towels. The procedure was repeated for all test materials separately. Materials were tested in random order. No more than two materials were tested in the same day and there was at least five hours interval between the tests.

A skin swab (back‐side of the right hand, 3 × 3 cm area, 9 wipes) was taken twice, just before exposure and immediately after drying hands with a paper towel. A cotton wool stick was first soaked in Tween^®^ 20, used in sampling and cut to a sterile polyethene sample tube. Using the above‐mentioned protocol, hands seemed clean through naked eye but the cotton used for taking the swab was seemingly darker after exposure than before exposure. We want to emphasize that this study did not handle the safety of not using soap in everyday life. Here, our purpose was only to test how microbial diversity on skin changes when various biodiverse materials are handled.

### 2.2 Experimental design: Exposure via fabric packets (experiment 2)

In the second experiment, two urban volunteers tested fabric packets filled with dried, crushed, and sieved *Sphagnum* moss (particle size less than 1 mm). A layer of the material was placed inside a fabric packet of size 10 × 10 cm. Three different types of fabrics were tested: airlaid material ST047DIA (thickness 0.44 mm; SharpCell, Kausala, Finland), airlaid material DS100 (thickness 0.85 mm; SharpCell, Kausala, Finland), and cotton fabric (Marimekko, Helsinki, Finland).

Both volunteers tested one packet made of ST047DIA and one packet made either of DS100 or cotton fabric. The packets were placed on the inner forearms of the volunteers. Packets were tied on with a clean disposable self‐adhesive bandage (Pharmacare Sport Bandage). The volunteers were exposed to the packets for 3 hr and 45 min. Skin swabs (5 × 5 cm area, 10 s) were taken just before placing the packets and immediately after they were removed. A stick with a cotton wool tip was first wetted in Tween^®^ 20, used in sampling and placed into a sterile polyethylene sample tube. We want to point out, that our experimental set up does not take into account the possible disturbance caused by the packet itself. However, as the exposure time is short, we believe that the disturbance effect is low compared to the transfer of microbes.

Bacterial composition on skin before and after use of the packets was compared to the bacterial composition in the *Sphagnum* moss that was used as a raw material for the packets.

Experimental protocol was approved by the ethics committee of Tampere University Hospital (case number: ETL R15081). The study was carried out in accordance with the relevant guidelines and regulations in Finland and the volunteer's signed informed consents.

### 2.3 Sample preparation for MiSeq sequencing

Skin swab samples were stored in deep freezer (<−70°C) in tubes containing Tween^®^ 20 (MP Biomedicals) (0.1%) + NaCl (0.1 mol/L, J.T.Baker) before DNA extraction. Total DNA was extracted from samples using PowerSoil^®^ DNA Isolation Kit (MoBio Laboratories, Inc., Carlsbad, CA, USA) according to the manufacturer's standard protocol. The swab was transferred to the PowerBead tube for homogenization and lysis. For the moss sample used in experiment 2, approximately 0.25 g of moss was used for DNA extraction. DNA was checked with agarose gel (1.5%) electrophoresis. Total DNA concentration was measured with Quant‐iT^™^ PicoGreen^®^ dsDNA reagent kit (Thermo scientific, MA, USA).

In experiment 1, DNA was analyzed for bacterial (16S) communities using a two‐step PCR approach to avoid a 3′‐end amplification bias resulting from the sample‐specific DNA tags (Berry, Mahfouldh, Warner, & Loy, 2011). The variable regions 1–3 within the 16S ribosomal RNA (rRNA) gene was amplified by primary PCR (three replicates from each sample) using pA and PD Illumina primers with overhangs. Primary PCR was carried out in a reaction mixture (reaction volume 50 μl) consisting of 1 μl each of 10 mmol/L deoxynucleotide triphosphates (dNTPs; Thermo scientific, MA, USA), 5 μl forward primer pA_Illum_FP (10 μmol/L; ATCTACACTCTTTCCCTACACGACGCTCTTCCGATCTAGAGTTTGATCMTGGCTCAG) and 5 μl reverse primer pD′_Illum_RP (10 μmol/L; GTGACTGGAGTTCAGACGTGTGCTCTTCCGATCTGTATTACCGCGGCTGCTG), 0.5 μl 2 U/μl Phusion Green Hot Start II High‐Fidelity DNA polymerase (Thermo scientific, MA, USA), 10 μl 5× Green HF PCR buffer (F‐537), 5 μl template DNA, and 23.5 μl sterile water. The PCR reaction was performed in a thermocycler (MJ Research, MA, USA) as follows: initial denaturation at 98°C for 5 min, followed by 30 cycles with denaturation at 94°C for 1 min, annealing for 10 s at 50°C and extension for 1 min at 72°C, and then a final extension at 72°C for 10 min. A positive control (*Cupriavidus necator* JMP134, DSM 4058) was included in PCR runs and a negative control (sterile water) was run to detect any possible contamination. DNA was detected with agarose gel (1.5%) electrophoresis. The PCR products were purified using Agencourt AMPure XP solution (Beckman Coulter Ins.) to reduce carryover of primary PCR primers. Illumina adapter overhang nucleotide sequences were added to the 16S rRNA gene‐specific sequences in the secondary PCR. The secondary PCR and sequencing were performed at The Institute of Biotechnology (University of Helsinki) using Illumina MiSeq platform. In the secondary PCR, full length adapters and Indexes were introduced. The PCR protocol was as described by Koskinen, Hultman, Paulin, Auvinen, and Kankaanpää (2011). The sequencing was done as paired‐end (300 bp+300 bp) on a MiSeq Illumina instrument using a v3 reagent kit.

In experiment 2, the V4 region within the 16S ribosomal RNA (rRNA) gene was amplified by primary PCR as triplicates using 505F and 806R primers (Caporaso et al., 2012). Primary PCR was carried out in a reaction mixture (reaction volume 50 μl) consisting of 1 μl each of 10 mmol/L dNTPs (Thermo scientific, MA, USA) 5 μl forward primer 505F (10 μmol/L; 5′–GTGCCAGCMGCCGCGGTAA‐3′) and 5 μl reverse primer 806R (10 μmol/L; 5′–GGACTACHVGGGTWTCTAAT‐3′), 0.5 μl 2 U/μl Phusion Green Hot Start II High‐Fidelity DNA polymerase (Thermo scientific, MA, USA), 10 μl 5× Green HF PCR buffer (F‐537), 5 μl template DNA and 23.5 μl sterile water. The PCR reaction was performed in a thermocycler (MJ Research, MA, USA) as follows: initial denaturation at 98°C for 5 min, followed by 30 cycles (only 25 cycles was used for the moss sample) with denaturation at 94°C for 1 min, annealing for 10 s at 50°C and extension for 1 min at 72°C, and then a final extension at 72°C for 10 min. A positive (*Cupriavidus necator* JMP134, DSM 4058) and a negative controls (sterile water) were included in PCR and DNA was detected with agarose gel electrophoresis. The PCR products were purified using Agencourt AMPure XP solution (Beckman Coulter Ins.). Triplicates of the cleaned amplicons were pooled and diluted 1:5. Cleaned and diluted primary PCR products were targeted in the secondary PCR (TagPCR). Reaction mixture to the TagPCR was equal as above except reverse primer included a 12 bp unique Multiplexing Identifier tag (MID‐806R). Amplification program was the same as above except only ten cycles were used (7 cycles for the moss sample). TagPCR products were detected on agarose gel electrophoresis, purified with Agencourt AMPure, pooled and DNA concentration was measured with PicoGreen. The sequencing was conducted at the Kansas State University using Illumina MiSeq platform with a 2 × 300 bp version 3 sequencing kit according to manufacturer's protocol and the GeneRead DNA Library I Core Kit (Qiagen, catalog # 180432) was used to ligate Illumina's TruSeq adapters to amplicons.

### 2.4 Sequence processing

Raw sequencing data were processed using Mothur‐program (versions 1.36.1 and v.1.38.1). The datasets for experiments 1 and 2 were processed separately although using almost the same sequence processing protocol. The protocol partly followed the pipeline suggested by Schloss, Gevers, and Westcott (2011) and Kozich, Westcott, Baxter, Highlander, and Schloss (2013). The paired sequences in reverse and forward fastq files were aligned into contigs. Sequences were trimmed and screened to remove any mismatches with primer or DNA‐tag sequences, ambiguous bases and homopolymers larger than 8 bp long. Sequences were aligned using Mothur version of SILVA bacterial reference sequences (version 132) (Pruesse et al., 2007) and the sequences which were not aligned to a reference alignment of the correct sequencing region were removed. Unique sequences and their frequency in each sample were identified, and then, almost identical sequences (>99% similar) were preclustered to minimize sequencing errors (Huse, Welch, Morrison, & Sogin, 2010) and screened for chimeras with UCHIME (Edgar, Haas, Clemente, Quince, & Knight, 2011) which uses the abundant sequences as a reference. The chimeric sequences were removed. We calculated a pairwise distance matrix for unique sequences and clustered OTUs at 97% sequence similarity using the nearest neighbor algorithms. Sequences were classified using the Mothur version of Bayesian classifier (Wang, Garrity, Tiedje, & Cole, 2007) with the RDP training set version 16 (Cole et al., 2009). Sequences classified to Chloroplast, Mitochondria, unknown, Archaea, and Eukaryota were removed from the analyses. Rare OTUs that were represented by 10 or fewer sequences in the whole data were removed as suggested by Oliver, Brown, Callaham, and Jumpponen (2015). For each OTU the number of sequences found in negative controls were subtracted from each sample. This kind of procedure was a compromise between two extremes: removing all OTUs found in controls and not removing any. It has been suggested not to remove OTUs identified in negative controls if they are biologically expected (Salter et al., 2014). In this study, it was very difficult to identify biologically expected bacteria because bacteria could originate from human skin, soil or plant material. On the other hand, totally ignoring the OTUs found in negative controls might have resulted in relatively large percentage of false OTUs in before samples as these samples had much lower DNA concentration compared to the after samples (Figure 3.). Finally, all the samples were rarefied to 7,286 sequences in experiment 1 and to 11,511 sequences in experiment 2, which were the lowest number of sequences in each experiment.

### 2.5 Quantitative PCR

In experiment 1, we used the quantitative PCRs (q‐PCRs) of bacterial 16S rRNA gene based on SYBR green detection. PCRs were carried out with the Light Cycler 96 Quantitative real‐time PCR machine (MJ Research, MA, USA). The forward primer used was pE 5′‐AAA CTC AAA GGA ATT GAC GG‐3` and the reverse primer pF 5′‐ACG AGC TGA CGA CAG CCA TG‐3` (Kanto Öqvist et al., 2008). All samples were amplified in triplicates in 20 μl reactions containing 10 μl 2× PowerUp SYBR Green Master Mix (Thermo scientific, MA, USA), 0.2 μl 20 mg/ml BSA, 0.5 μl of each primer (10 μmol/L), and the sample template. A standard curve was included in every run to allow quantification of the number of bacterial 16S copies present in the original sample. The q‐PCR cycling was as follows: initial denaturation at 95°C for 2 min, followed by 40 cycles of denaturation at 95°C for 10 s, annealing for 20 s at 53°C and extension for 30 s at 72°C. Melting curve analysis on the amplicon was as follows: 95°C for 10 s, 65°C for 60 s, 97°C for 1 s, 37°C for 30 s with continuous measurement of the fluorescence signal. DNA of *Cupriavidus necator* JMP134 (DSM 4058) was used as a standard that worked also as a positive control while sterile water was used as a negative control.

### 2.6 Statistical methods, experiment 1

For experiment 1, the analyses were conducted for the whole community at different taxonomic levels (i.e., OTU, genus, family, order, class, and phylum). At OTU level, analyses were also conducted within phyla Acidobacteria, Actinobacteria, Bacteroidetes, Firmicutes and Proteobacteria and classes Alpha‐, Beta‐, Gamma‐ and Deltaproteobacteria. This kind of approach is sometimes called as deconstructing species diversity and it means that the whole community data are partitioned into smaller groups for example, by taxon, guild or other grouping (Marquet, Fernández, Navarrate, & Valdovinos, 2004; Tolonen et al., 2016). We used this approach because finer taxonomic groupings may show patterns differing from the patterns found for the whole community (Marquet et al., 2004). This commonly used approach has previously been used also with PERMANOVA and PERMDISP (Anthony, Frey, & Stinson, 2017; Grönroos et al., 2013).

Paired Wilcoxon signed‐rank test or Student's *T*‐test was used for comparing the number of bacterial 16S copies, number of bacterial OTUs (i.e., richness) and Shannon diversity index of the bacterial community in hands before and immediately after exposure. *T*‐test was conducted when the data were normally distributed based on Shapiro‐Wilk test and Wilcoxon signed‐rank test was conducted when the data were not normally distributed.

The difference between bacterial composition in hands before and immediately after exposure was studied using Permutational Multivariate Analysis of Variance (PERMANOVA) (Anderson, 2001), Multivariate Homogeneity of Group Dispersions (PERMDISP) (Anderson, 2006; Anderson, Ellingsen, & McArdle, 2006) and visually illustrated using Non‐Metric Multidimensional Scaling (NMDS). PERMANOVA was run using two factors: before/after treatment and person. PERMDISP was run using bias correction (Stier, Geange, Hanson, & Bolker, 2013) and spatial median as the group centroid. In all the three methods, Bray‐Curtis distance was used. PERMANOVA and PERMDISP were run using 999 permutations. PERMANOVA, PERMDISP, and NMDS were not conducted for Acidobacteria and Deltaproteobacteria because several samples did not contain any bacteria belonging to these two taxa. For Bacteroidetes, one sample included only one sequence which masked the differences among all the other samples. Thus, for Bacteroidetes, the given sample pair was removed when running NMDS, PERMANOVA, and PERMDISP.

Analyses were done using R (version 3.3.3) and package *vegan* (version 2.4‐3) (Oksanen et al., 2017).

### 2.7 Statistical methods, experiment 2

Number of OTUs and Shannon diversity index were calculated using functions specnumber and diversity in R package *vegan,* respectively. Principal Coordinate Analysis was performed using cmdscale function in R package *stats* and it was based on Bray‐Curtis distance calculated for both abundance and presence‐absence data and using function vegdist in package *vegan*.

## 3 RESULTS

### 3.1 Direct hand‐exposure

**Figure 1:**
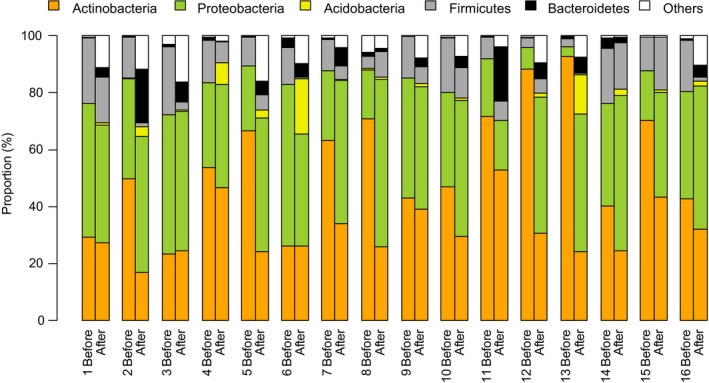
Relative abundances of the five most abundant phyla in skin swab samples before and immediately after exposure to plant and soil materials (experiment 1). Each of the two volunteers tested eight materials. In total, 16 different materials were tested. Each bar shows results for one individual sample

**Figure 2:**
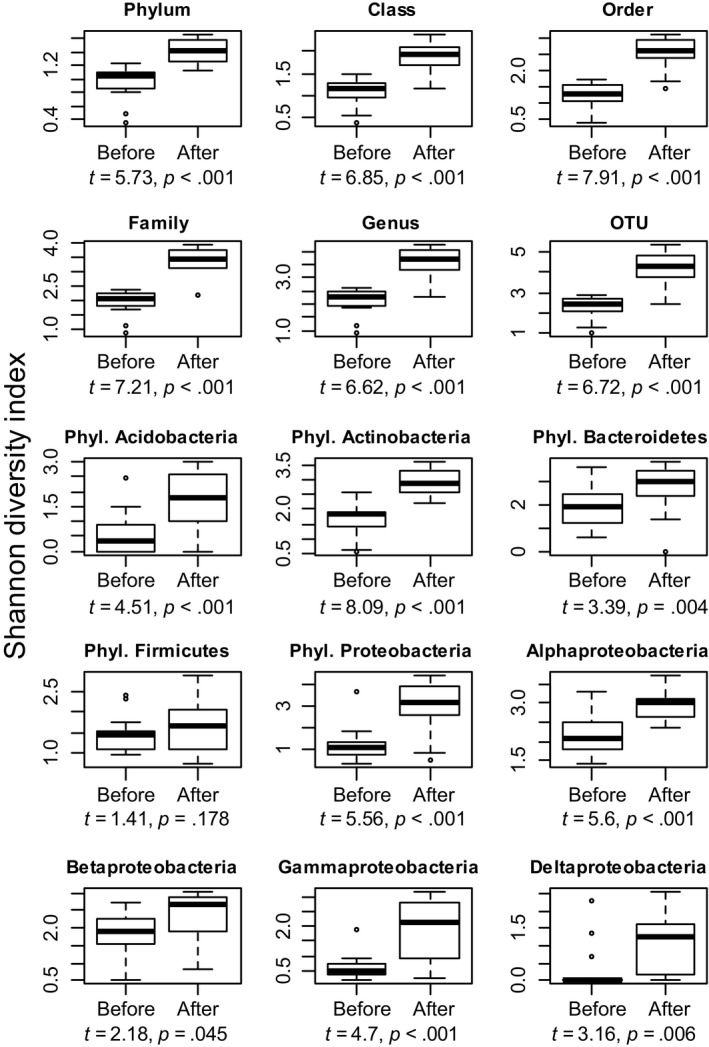
Shannon diversity of bacteria in hands increased after exposure to plant and soil materials (experiment 1). Results are shown for the whole data at six different taxonomic levels (phylum, class, order, family, genus and OTU) as well as for OTUs within five major phyla (Acidobacteria, Actinobacteria, Bacteroidetes, Firmicutes, and Proteobacteria) and four classes within phylum Proteobacteria. Boxplots show medians (thick line), upper and lower hinges (box), minimum and maximum values (whiskers) and outliers (points; values more than 1.5 times the interquartile range from the hinges). Data were normally distributed based on Shapiro‐Wilk test and thus paired *T*‐test was used. Degrees of freedom is 15 for all tests (*n* = 16, each of the two volunteers tested eight materials)

In the first experiment, two urban volunteers tested eight soil and plant based materials by rubbing the materials in their hands. Altogether sixteen different materials were tested. Each material was tested separately and skin swab sample was taken before and after the exposure. Illumina MiSeq sequencing of bacterial 16S RNA gene showed that the most common bacterial phyla in hands before the exposure were Actinobacteria, Proteobacteria, and Firmicutes (Figure 1). After the exposure, the relative abundance of Acidobacteria and Bacteroidetes increased. The relative abundance of several less common phyla increased (group “others” in Figure 1), which also led to an increase in Shannon index and richness for the phyla (Figures 2 and S1).

**Figure 3:**
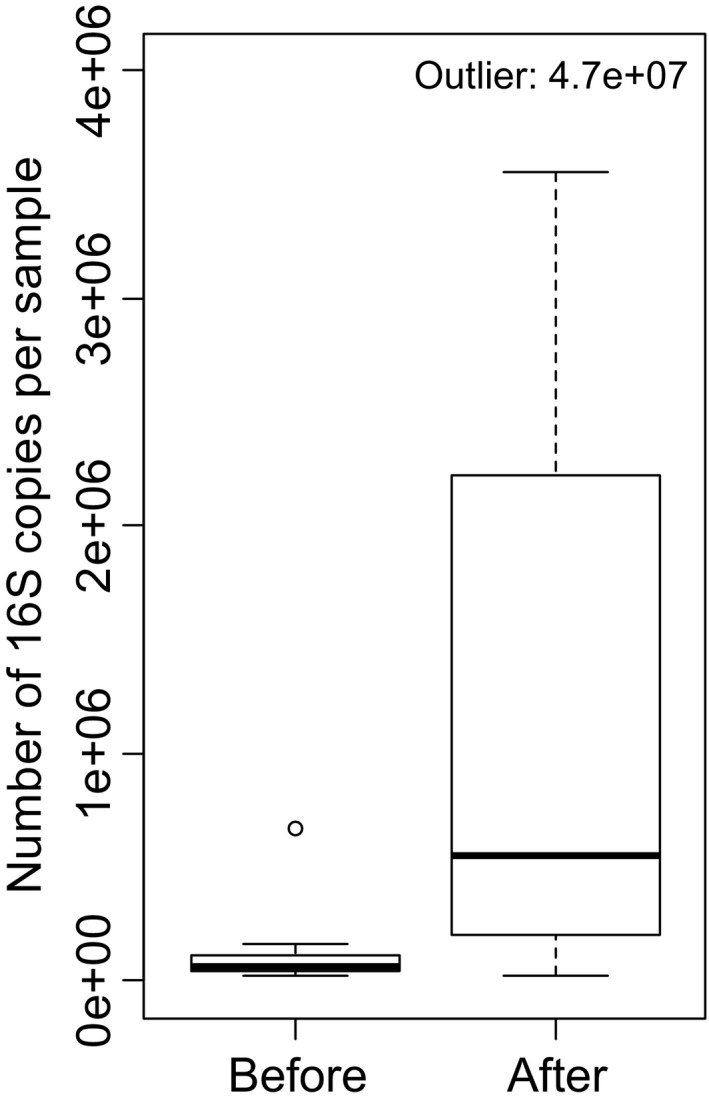
Quantitative PCR shows increase in bacterial abundance in hands after exposure to soil and plant based materials (experiment 1). Sixteen materials were tested and quantitative PCR was conducted for samples taken before (*n* = 16, each of the two volunteers tested eight materials) and after (*n* = 16) each exposure. For visual reasons, one outlier in after samples is left outside the axis margins. Paired Wilcoxon signed‐rank test for whole data is *p* = .001 and *V* = 9. For data without the pair with the outlier is *p* = .002 and *V* = 9. See boxplot description in Figure 2

Shannon diversity index (Figure 2) and taxon richness (i.e., number of taxa, Figure S1) were significantly higher (*p* ≤ .045) after exposure at all tested taxonomic levels as well as for all tested taxonomic groups except for the phylum Firmicutes. Quantitative PCR and Wilcoxon signed‐rank test also showed that the total bacterial abundance (i.e., number of bacterial 16S copies) was significantly higher after exposure compared to that before exposure (*p* = .001, *V* = 9) (Figure 3).

**Figure 4:**
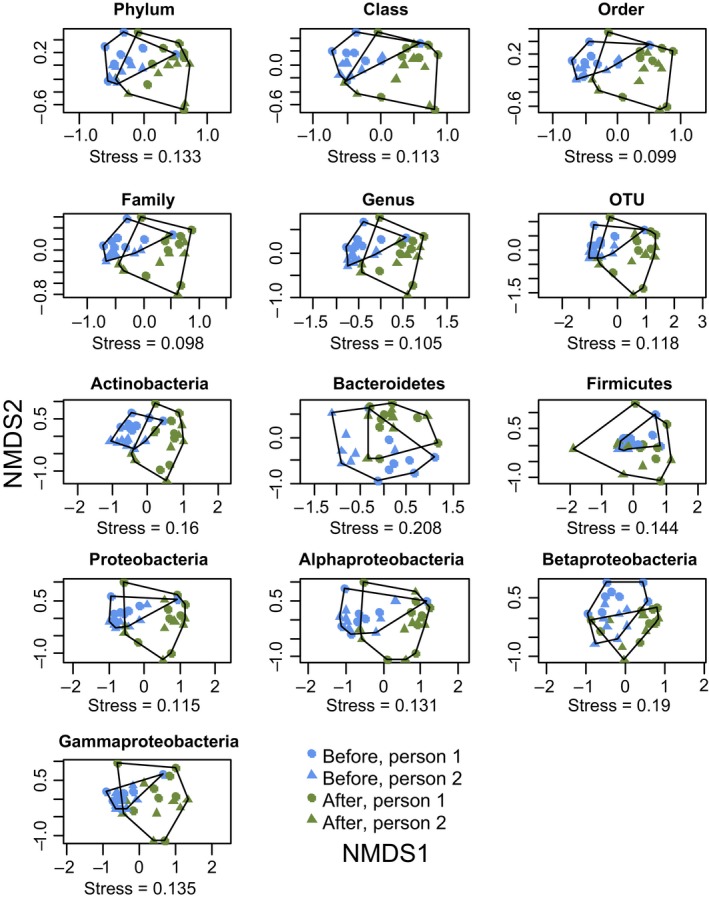
Bacterial community composition in hands changed after exposure to plant and soil materials (experiment 1). Non‐Metric Dimensional Scaling ordinations for bacterial community composition at six different taxonomic levels (phylum, class, order, family, genus, and OTU) as well as for OTUs within four major phyla and three classes within phylum Proteobacteria (*n *=* *16, each of the two volunteers tested eight materials)

Bacterial community composition in hands was significantly different after exposure compared to that before exposure at all tested taxonomic levels and in all tested taxonomic groups (Table 1, Figure 4). Permutational Multivariate Analysis of Variance (PERMANOVA) showed that the effect of exposure treatment (before/after) was highly significant. The identity of person was also significant in many cases in PERMANOVA analysis, but the interaction between treatment and person was significant only at the phylum level (*p* = .017) and for the phylum Actinobacteria (*p* = .011). The lack of interaction suggests that the change in community composition did not depend on the person conducting the experiment. We also plotted NMDS figure with the sampling order (Figure S2). This showed that although the materials were tested consecutively (minimum of 5 hr between two test occasions), in most cases, the skin bacterial composition had recovered between the sampling occasions.

**Table 1:** Bacterial community composition in hands changed after exposure to plant and soil based materials (experiment 1)

	PERMANOVA									PERMDISP			
	*p*‐value			*F*			*r* ^2^			*p*‐value	*F*	Mean bef	Mean aft
	Bef/Aft	Person	Interact.	Bef/Aft	Person	Interact.	Bef/Aft	Person	Interact.				
Taxonomic levels													
Phylum	**0.001**	**0.006**	**0.017**	19.88	8.34	5.38	0.32	0.14	0.09	0.501	0.53	0.19	0.16
Class	**0.001**	**0.004**	0.055	18.32	6.29	2.65	0.33	0.11	0.05	0.197	1.74	0.20	0.25
Order	**0.001**	**0.009**	0.093	18.31	4.92	2.03	0.34	0.09	0.04	**0.012**	7.01	0.21	0.32
Family	**0.001**	**0.001**	0.093	14.80	5.81	2.08	0.29	0.11	0.04	**0.035**	4.60	0.30	0.41
Genus	**0.001**	**0.003**	0.086	13.22	5.65	1.89	0.27	0.12	0.04	**0.029**	5.42	0.32	0.44
OTU	**0.001**	**0.002**	0.085	8..68	5.55	1.78	0.20	0.13	0.04	**0.001**	11.74	0.34	0.51
OTU level within Phyla/Class													
Actinobacteria	**0.001**	**0.001**	**0.011**	9.63	7.39	2.97	0.20	0.15	0.06	**0.02**	6.77	0.38	0.49
Bacteroidetes	**0.001**	**0.007**	0.524	2.21	1.69	0.95	0.07	0.05	0.03	0.145	2.18	0.63	0.67
Firmicutes	**0.009**	**0.005**	0.394	4.36	4.83	0.90	0.11	0.13	0.02	**0.048**	4.55	0.30	0.45
Proteobacteria	**0.001**	**0.023**	0.536	8.45	3.18	0.77	0.21	0.08	0.02	**0.006**	8.32	0.31	0.49
Alphaproteobacteria	**0.001**	0.693	0.861	9.10	0.71	0.59	0.24	0.02	0.02	0.944	0.01	0.49	0.49
Betaproteobacteria	**0.001**	**0.003**	0.473	4.48	2.53	0.95	0.12	0.07	0.03	0.745	0.11	0.59	0.57
Gammaproteobacteria	**0.002**	**0.009**	0.492	4.34	3.71	0.86	0.12	0.10	0.02	**0.006**	9.50	0.29	0.51

Results of permutational multivariate analysis of variance (PERMANOVA) with two factors (before/after, study person and their interaction) and results of the analysis of multivariate homogeneity of group dispersions (PERMDISP). Bray‐Curtis dissimilarity was used. Analyses were not done for phylum Acidobacteria and class Deltaproteobacteria because several samples did not include any bacteria belonging to these two taxa. For PERMDISP, also the mean values for the distances to group medians are shown. Each of the sixteen materials was tested once by one of the two study participants. *P*‐values less than 0.05 are highlighted in bold.

Finally, Multivariate Homogeneity of Group Dispersions (PERMDISP) showed that the variation in bacterial composition was significantly higher after exposure compared to the variation before exposure for the whole data at order, family, genus, and OTU levels as well as for OTU level data within Actinobacteria, Firmicutes, Proteobacteria, and Gammaproteobateria (Table 1, Figure 4).

### 3.2 Exposure via fabric packets

In the second experiment, two urban volunteers used moss‐filled fabric packets on their skin. Bacterial richness and Shannon diversity index were higher in samples taken after the use of packets containing the *Sphagnum* moss compared to those taken before the use of the packets except for the richness of ST047DIA_2 (Table S1). Principal Coordinate Analysis showed that after the experiment, the bacterial community composition on skin was different from the samples taken before the experiment (Figure S3). In addition, after the exposure, the skin bacterial composition was more similar to the sample of moss material than before the exposure suggesting that some bacterial OTUs were transferred from the packets to the skin.

## 4 DISCUSSION

While previous efforts have shown that microbes can be transplanted from one body site to the other (e.g., Costello et al., 2009), we show here that also microbes in natural materials can be attached to skin to increase its microbial diversity. The reasoning was that the vicinity of diverse microbial communities has been suggested to reduce the risk of immune‐mediated diseases (von Hertzen et al., 2011). The immediate response that we found is relevant as even a transient change is plausibly of utmost importance at times of dining and touching lips or nostrils by hand. One potential way to affect microbial exposure of other organs is to modify skin microbiota of hands. Here, we exposed hands with soil and plant materials and observed a drastic increase in skin microbial diversity.

Until now, approaches to cure or prevent immune system disorders using microbe‐based treatments have been limited to the use of living probiotic bacteria (Abrahamsson, Jakobsson, Björkstén, Oldaeus, & Jenmalm, 2013; Marschan et al., 2008), inactivated bacteria (Berth‐Jones et al., 2006; Brothers, Asher, Jaksic, & Stewart, 2009), bacterial parts (Kline, 2007), bacterial or helminth excretions (McSorley et al., 2012) and fecal transplantations. Of these methods, only fecal transplantation has been proven to be effective but it is restricted to only a few diseases, such as severe *Clostridium difficile* diarrhea and inflammatory bowel diseases, and it is hardly suitable for treatments aimed at modulating immune system to prevent immune‐mediated diseases (Cohen & Maharshak, 2017). Other approaches are based on exposure to certain microbes, such as certain bacterial species, their components or excretions, to modulate immune system. Some studies have found these methods promising (Kline, 2007; Marschan et al., 2008; McSorley et al., 2012) while others have found no effects (Abrahamsson et al., 2013; Berth‐Jones et al., 2006; Brothers et al., 2009). Of these methods, use of probiotics has received substantial amount of research interest and several studies have shown that, when applied to infants or during pregnancy, they reduce the risk of atopy and allergy, although contradictory results have also been reported (Nermes, Salminen, & Isolauri, 2013).

In the current study, we substituted the strain‐based approach with nature‐based materials comprising diverse microbial communities. We found a pronounced increase in diversity of skin microbiota immediately after short‐term direct exposure of hands to natural soil and plant based gardening materials. Also, the total abundance of bacteria was higher after exposure than before exposure. These results indicate that bacteria in soil and plant materials are attached to skin and remain there even after washing hands with water. In the second experiment, we tested a more convenient way of applying the exposure material and found that skin microbial diversity also increased when using fabric packets filled with dried and crushed moss. Together, these results suggest that introducing an increased exposure to nature‐based materials has a potential to transiently change the skin microbiota. These findings open new possibilities to study exposures that could be used to modulate functions of the immune system, particularly if the timing of the exposure is optimized.

In previous studies, contradicting results when using approaches based on single or a few microbial species, might be due to a mismatch between individual disease phenotype and the microbial species or strain used (Abrahamsson et al., 2013; Nermes et al., 2013; Stiemsma et al., 2015). Thus, using materials with natural diversity, such as the soil and plant based materials tested here, might be a more effective approach to enhance microbial diversity and therefore prevent and cure immune system disorders. The approach presented here simulates working in garden or in the field—practices previously common in human everyday life but nowadays largely lacking from lives of urban citizens. In addition, with the exposure strategy presented herein, the immune system encounters environmental microbial stimuli in a very natural fashion. We assume that this kind of exposure has a potential to stimulate the whole immune system including different anatomic locations such as skin, oral and respiratory mucosa, Peyer's patches, cilia in gastrointestinal tract, digestive enzymes, and finally, the exposure interact with different lymphocytic cells. Indeed, several studies have shown a relationship between diversity of human microbiota and reduced risk of immune‐mediated diseases (Abrahamsson et al., 2012; Hanski et al., 2012; Manichanh, 2006; Scher et al., 2015) and rural living environment has been shown to be positively related to the diversity of human microbiota (De Filippo et al., 2010; Schnorr et al., 2014; Yatsunenko et al., 2012). It is likely, that the true mechanism behind these patterns is not a single bacterial species or a strain, but rather, a higher exposure to naturally diverse microbiota.

Our results showed that touching natural soil and plant based materials increased, at least temporarily, the total diversity of skin microbiota and the diversity of phyla Acidobacteria, Actinobacteria, Bacteroidetes, Proteobacteria as well as classes Alpha‐, Beta‐ and Gammaproteobacteria. We also found that for many groups the variation in community composition (i.e., beta diversity) was higher immediately after the exposure. Because several different materials were used in the exposure, it might be possible to mix different natural soil and plant materials and thus produce a composite material that would lead even to a higher increase in skin microbial diversity. Our results also showed that the skin microbiota recovered quickly close to the initial composition. This suggests that single exposure may not be enough to produce long‐lasting effects and thus repeated exposures might be needed.

The approach presented here, is, at least initially, concentrating on modifying skin microbiota. Diverse skin microbiota can have direct beneficial effects on human health (Rodrigues Hoffmann, 2017). For example, commensal microbes inhibit growth of pathogenic microbes by competing for nutrients and space, thus reducing the growth of pathogens (Sanford & Gallo, 2013). In addition, a large number of commensal bacteria directly restrict the growth of competitors through the production of antimicrobial compounds (Gallo & Nakatsuji, 2011) and others are able to amplify the innate immune response of human keratinocytes to pathogens (Wanke et al., 2011). In addition, the increased microbial diversity on skin may affect also stool microbiota as microbes are potentially transferred from hands to gastrointestinal tract through hand contacts with respiratory and oral mucosa and with food. The high importance of balanced stool microbiota on human well‐being and immune system has been suggested in a number of studies (Abrahamsson et al., 2012; Hartstra, Nieuwdorp, & Herrema, 2016; Manichanh, 2006).

We have shown here that direct exposure to natural soil and plant based materials is a potential approach to change skin microbiota. Based on these results and because a recent consumer survey indicated that many consumers would be willing to buy health‐enhancing products (Puhakka, Valve, & Sinkkonen, 2017), we propose that microbial exposure of urban dwellers can be increased by designing touchable plant and soil based consumer products comprising rich microbial flora. This study presents the first test and thus further studies on the effects of various nature‐based exposures are needed. Future studies need to examine (1) how long the change in skin microbiota is preserved, (2) does the exposure translate into changes in gut microbiota, and (3) if the exposure induces beneficial changes in the immune system markers such as cytokines and Th1/Th2 balance. We conclude that, in addition to ongoing research on probiotics and other approaches to prevent or cure immune mediated diseases, more research is needed on possibilities offered by the nature‐based approaches such as those presented in this paper. Our research group is currently examining the above questions and developing techniques for producing natural exposure materials suitable for everyday use (Nurminen et al., 2018).

## CONFLICT OF INTEREST

Submitted paper is related to a patent application (Applicants: University of Helsinki and University of Tampere; Inventors, among others: MG, OHL, HH, and AS) .

## DATA AVAILABILITY

Raw sequence reads are available in Sequence Read Archive with accession numbers SRP111440 and SRP113302. The datasets generated during and/or analyzed during the current study are available from the corresponding authors on reasonable request.
