# Supplementary material for: Short‐term direct contact with soil and plant materials leads to an immediate increase in diversity of skin microbiota
Source: Microbiologyopen. 2018 May 29;8(3):e00645. doi: 10.1002/mbo3.645 (PMC6436432; doi:10.1002/mbo3.645)
Supplement: Supplementary file 1 [file MBO3-8-e00645-s001.docx]

Supporting Information

**Title:** Short-term direct contact with soil and plant materials leads to an immediate increase in diversity of skin microbiota

**Authors:** Grönroos, M., Parajuli, A., Laitinen, O.H., Roslund, M., Vari, H., Hyöty, H., Puhakka, R. & Sinkkonen, A.

**Contents of the supporting information**

**Fig S1. Page 2:** Bacterial richness (number of OTUs) in hands increased after exposure to plant and soil materials. (experiment 1)

**Fig S2. Page 3:** Bacterial community composition in hands before and after exposure to plant and soil materials. (experiment 1)

**Fig. S3. Page 6:** Bacterial community composition on skin changed after exposure to moss material. Principal Coordinate Analysis for skin swab samples taken before (dark grey) and after (black) use of fabric packets filled with crushed and air dried moss as compared to original moss material (light grey). (experiment 2)

**Table S1. Page** **7**: Bacterial richness and diversity in hands increased after exposure to packets filled with moss material. Two participants tested two packets made of different textile (ST04DIA was tested by both participants). Values before and after using the packets are shown. Also the respective values in moss material used inside the packets are shown. (experiment 2)

**Fig. S1.** Bacterial richness (number of OTUs) in hands increased after exposure to plant and soil materials. Results are shown for the whole data at six different taxonomic levels (phylum, class, order, family, genus and OTU) as well as for OTUs within five major phyla (Acidobacteria, Actinobacteria, Bacteroidetes, Firmicutes and Proteobacteria) and four classes within phylum Proteobacteria. Boxplots show medians (thick line), upper and lower hinges (box), minimum and maximum values (whiskers) and outliers (points; values more than 1.5 times the interquartile range from the hinges). Data were normally distributed based on Shapiro-Wilk test and thus paired T-test was used. Degrees of freedom is 15 for all tests. (experiment 1)

**Fig S2 A.**

**Fig S2 B.**

**Fig S2.** Bacterial community composition in hands before (blue) and after (green) exposure to plant and soil materials. Non-Metric Dimensional Scaling ordinations are shown for bacterial community composition at six different taxonomic levels (phylum, class, order, family, genus and OTU) as well as for OTUs within four major phyla and three classes within phylum Proteobacteria. The same ordination scores are used here as in Fig. 4. in the main paper, but scores are plotted separately for study person 1 (A) and 2 (B). Additionally, the sampling order is given inside the points and line is drawn to connect the successive samples. (experiment 1)

**Fig S3.** Bacterial community composition on skin changed after exposure to moss material. Principal Coordinate Analysis for skin swab samples taken before (dark grey) and after (black) use of fabric packets filled with crushed and air dried moss as compared to original moss material (light grey). (experiment 2)

**Table S1**. Bacterial richness and diversity in hands increased after exposure to packets filled with moss material. Two participants tested two packets made of different textile (ST04DIA was tested by both participants). Values before and after using the packets are shown. Also the respective values in moss material used inside the packets are shown. (experiment 2)

| Packet textile | Sample | Number of OTUs | Shannon index |
| --- | --- | --- | --- |
| DS100 | Before | 178 | 2.94 |
| DS100 | After | 228 | 3.36 |
| Cotton fabric | Before | 163 | 2.71 |
| Cotton fabric | After | 269 | 3.72 |
| ST047DIA_1 | Before | 194 | 2.60 |
| ST047DIA_1 | After | 236 | 3.16 |
| ST047DIA_2 | Before | 230 | 3.15 |
| ST047DIA_2 | After | 225 | 3.47 |
| Moss |  | 477 | 3.75 |
